# Cæsarean Operation on a Dwarf

**Published:** 1843-05

**Authors:** Cyrus Falconer

**Affiliations:** Hamilton, Butler county, Ohio


Art. IV.—*Cæsarean Operation on a Dwarf.* By Cyrus Falconer, M. D., of Hamilton, Butler county, Ohio.

Perfect success is not always the test of merit or skill; but it is sometimes the chief motive in reporting cases for the public eye. I am not positively sure that some influence of this kind has not prevented me from offering at an earlier date a report of the following case, which has certainly some peculiar and striking features, however barren of brilliancy in the result.

On the 6th of July, 1840, I was called to visit Miss —— ———, of Ross township, in the south-western part of this county (Butler), in labor with first child. The physician in attendance was Dr. Praether, an intelligent practitioner of Venice, near which village the patient resided. She had been in labor since the previous midnight; I arrived about 2½ P. M.

I was not a little surprised to find the patient a *dwarf*, just *three feet six inches in height*, with a form very illy proportioned. Her head was perhaps of normal size, and her trunk not greatly inferior in *breadth* to the ordinary standard, but longitudinally reaching little over its proportion of her diminutive stature. The left foot of the fœtus was presenting at the os externum, and Dr. P. informed me he had felt the toes of its fellow; but had been unable to introduce his hand so as to grasp the foot and bring it down. I proceeded to examine her; and carrying the finger to the superior strait, I immediately discovered a mal-conformation of the pelvis. The sacrum projected towards the pubis so as to give the superior strait the character of a *fissure;* the antero-posterior diameter being certainly not over one inch and three-fourths. The leg of the presenting foot occupied the full breadth of the fissure, affording an evidence but too conclusive that nature was not competent to the delivery. The os uteri was well dilated, and the pains incessant and severe. Carrying my hand over the abdomen I found the uterus occupying a diagonal position; the fundus extending high into the right hypochondriac region.

Her mother assured us that the mal-formation was congenital, and that she had observed the pelvic obliquity soon after birth: but from a history of the early childhood of the patient we were satisfied it was the result of *rachitis.*

What was to be done? The strength of our patient was flagging in an unavailing travail. Her delivery *per vias naturales,* was evidently impossible. Shall we sacrifice the fœtus in an attempt to save the mother? *Can* we, after awaiting its death, remove it piecemeal? These questions were rapidly and anxiously revolved in our minds. Had the head presented, its reduction and the use of the crotchet would of course have presented themselves, though had that been the case there was not room, I apprehend, for the passage of the base of the cranium. Under the circumstances, we were soon brought to the conclusion that the only hope for either child or mother was in the *Cæsarean section.* This *might* save both, without it the loss of both was inevitable; for we had not sufficient confidence in the division of the pubis to induce us to canvass its merits.

Quietly withdrawing the mother of the patient, and one of her friends, we stated to them frankly the situation of the case, and the alternatives. They were less surprised than I had anticipated, the dwarfish stature and disproportions of the girl having prepared them for something of the kind. We next communicated our views to the patient herself—she exhibited but slight emotion, and promptly agreed to the operation.

We then proposed sending to Hamilton—eight miles—for additional counsel: but to this the patient strongly objected, insisting she could not endure the delay, and imploring us to proceed at once to the operation. Her mother joined in the request, and indeed we were satisfied that every hours delay lessened the prospect of a favorable result. The most propitious time, according to all writers on this subject, I believe, had already long since passed.

The operation resolved upon, we set about preparing for it. Ligatures, adhesive strips, lint, compresses, and a broad bandage were arranged ready for use. The instrument selected was the common scalpel.

Placing the patient upon her back on the bed which she was finally to occupy, with the lower extremities partially flexed, and having the walls of the abdomen compressed by assistants, so as to fix the uterus and prevent the escape of the omentum or intestines, I proceeded to make the first incision. In order to make myself understood I will repeat that the fundus of the uterus extended high into the right hypochondrium, overlapping, and to a considerable extent, dislodging the liver from its position. It was necessary to make the incision somewhat oblique, beginning at the upper part, near the right margin of the *linea alba,* and crossing towards its centre in the descent towards the pubis. The usual direction is to commence below the umbilicus: but in this case the shortness of the abdomen made it imperative to begin considerably higher up, in order to get an opening large enough to extract the fœtus. This case seemed to me a wonderful illustration of the capacity of nature to adapt herself to circumstances, however straitened. The liver and stomach appeared to be crowded entirely out of their proper location, pressing of course, in turn, upon the diaphragm and other viscera, and yet the functions of animal life had been but little disturbed. With the first sweep of the knife, I divided the abdominal integuments to within an inch or an inch and a half of the pubis, exposing the aponeurotic expansion which forms the linea alba, the whole distance: this was then carefully divided, and the peritoneum presented itself. Making a small orifice in this latter membrane at the upper extremity of the incision, I inserted a couple of fingers and slightly elevating them divided it; the fingers acting as a director, and protecting the parts beneath. At this part of the operation, much difficulty was experienced in preventing the escape of the intestines. The uterus was opened, observing the same caution as with the peritoneum, and the fœtus was exposed, its back presenting to the incision. Although I began my incision considerably above the umbilicus, such was the relative size of the child that I found it impracticable to extract it, until I had extended the opening in each direction; approaching nearly to the cartilage of the lower true rib above, and the pubis below. During my efforts to accomplish the delivery, considerable extravasation took place. The relative size of child and mother can only be conceived by the reader, when he remembers the height of the mother—3½ feet—and learns that the child was about the ordinary size, weighing, by conjecture, from seven to eight pounds.

I at length succeeded, by grasping the thighs in elevating the breech, and delivered the child, as in a breech presentation; it soon cried lustily, and was separated from the cord. The uterus now contracted powerfully, the placenta was expelled, the extravasated blood removed as much as possible, and we then proceeded to dress the wound.

Four or five points of the interrupted suture were employed—long adhesive strips were applied between the sutures, leaving a space at the lower portion, for the escape of any discharge that might accumulate. A broad compress was next applied, and the whole covered with a broad firm bandage tolerably tight.

During the operation, the patient made very little complaint; she now said she felt very comfortable, and expressed much gratification at being relieved by an amount of suffering, so much less than she had apprehended. An anodyne was administered, and finding her at the end of a couple of hours still comfortable and inclined to rest, I left her, and rode home, solacing myself with the pleasing hope of a favorable result. Ere morning, however, inflammation began to be developed. On visiting her next day, I found the tongue white, the pulse quick and frequent, the abdomen swelled, tympanitic, and tender, great thirst, with all the evidences of a high degree of inflammation. Dr. Praether residing near her, saw her frequently; but it is not necessary to detail the treatment, which did not differ from that usually pursued in inflammation of the abdominal viscera. She died on the eighth.

The child did well, and is now a vigorous healthy and well formed little girl.

An additional link in the chain of sympathy excited in my bosom for the luckless subject of this notice, was the fact that she was the victim of a human fiend, *in the shape of her own uncle!*

A word as to the proper time of operating and I have done. Cæsarian section must necessarily be an exceedingly rare operation in any country, and more especially in the sparse population and well-formed pelves of *our* country. This infrequency, with the want of observation and experience which flows from it, will ever tend to produce hesitancy and indecision in the minds of the medical attendant, and will probably very often delay the operation until the most elegible time has gone by. The fear of censure will doubtless sometimes throw its weight into the same scale; for it is unfortunately true, that there are many in our profession who will find fault with the practice of a rival brother, whenever it is practicable to do so. In *this* case it has been said we were too precipitate, and should have had additional counsel. But in fact the delay already had, was very possibly a cause of the unfortunate result to the mother. The unfavorable termination of nearly all the earlier cases of Cæsarian operation in England, is attributed chiefly to the late period of labor when the operation was resorted to.*

The continental writers, amongst them *Graefe* and *Baudelocque*, unanimously assure us that the proper time for the operation is before the waters have been evacuated, and just so soon as the os uteri is sufficiently dilated to permit their free discharge. In this opinion Dewees concurs; and no doubt such is the proper course where election as to time is within the control of the attendant. *Sabatier* says, "There ought not to be too much delay, lest the patient's strength be exhausted, and the violent efforts of labor should bring on an inflammatory state of the parietes of the uterus."

March, 1843.

* Vide Hull's letter, Denman, *et al.*

